# Simulated Impacts of Juvenile Mortality on Gulf of Mexico Sturgeon Populations

**DOI:** 10.1100/tsw.2002.92

**Published:** 2002-01-31

**Authors:** William B. Tate, Mike S. Allen

**Affiliations:** University of Florida, Department of Fisheries and Aquatic Sciences, 7922 NW 71st Street, Gainesville, FL 32653, USA

**Keywords:** long-lived species, conservation, population viability, endangered species, population modeling

## Abstract

We used an age-structured computer model to assess the impact of changes in juvenile mortality on the Gulf of Mexico sturgeon population in the Suwannee River, Florida. We simulated population trends under four levels of annual juvenile mortality (20, 25, 30, and 35%). As the rate of mortality increased, population size decreased, and rates of population growth shifted from positive to negative. Our models indicated that juvenile survival is important to the success of gulf sturgeon populations, and mortality estimates are needed to predict population viability. We suggest that life history studies in estuaries should be conducted, and bycatch rates for commercial fisheries should be quantified to aid in the management and conservation of gulf sturgeon.

## INTRODUCTION

The importance of subadult survival to populations of long-lived marine species has been stressed for marine turtles[1], fishes[2,3,4], and mammals[5]. Slight changes in mortality during juvenile and subadult stages may have relatively large effects on population growth[1,2]. For many long-lived marine species, managers can do little to alter natural mortality rates, though anthropogenic mortality, in the form of bycatch, can be curbed or prevented.

The Gulf of Mexico sturgeon, *Acipenser oxyrhinchus desotoi* (gulf sturgeon), is a long-lived, anadromous fish that once ranged from Tampa Bay, Florida to the lower Mississippi River[6]. An extensive commercial fishery led to declines in stocks during the 20$^{th}$ century[6]. A moratorium was placed on fishing in 1983[7], and this subspecies was listed as federally threatened in 1991[8]. Gulf sturgeon migrate into coastal rivers in late winter and early spring to spawn, inhabiting these rivers until late fall[6]. There is a downriver migration in October–November, and after a period of staging at the river mouth, subadults (age 4 to 12) and adults (age > 12) move into the Gulf of Mexico[6,7,9,10,11,12,13]. These fish inhabit nearshore mesohaline estuaries until water temperatures drop in mid-December, at which time they move into deeper water[12]. Due to sampling constraints (e.g., large mesh nets used in population surveys), life history information for gulf sturgeon less than 75 cm (approximate age = 3 years) is lacking. However, we know that juvenile gulf sturgeon (age 1 to 3) remain in the river-mouth estuary throughout the winter[12]. While inhabiting estuaries, gulf sturgeon may be incidentally captured as bycatch during commercial fishing[9], and juvenile fish may be more susceptible to capture than adults due to their small size and limited distribution. Because slight changes in survival of juvenile gulf sturgeon may affect population growth and viability, understanding the importance of this life stage is essential to conservation and restoration of this sub species. We evaluated how changes in juvenile mortality would influence gulf sturgeon population growth.

## METHODS

We simulated the impact of various levels of juvenile mortality on gulf sturgeon in the Suwannee River, Florida using the computer program MOCPOP 2.0[14]. We used an age-structured model based on published studies of gulf sturgeon (e.g., Table 1). However, length-fecundity estimates from white sturgeon, *A. transmontanus*[15], were used because estimates for gulf sturgeon or Atlantic sturgeon, *A. o. oxyrhinchus*, were unavailable. The age-specific number of fish in the population was calculated from the equation:

$$N_{(x+1,\ t+1)} = (N_{x,t})(S_x) \qquad (1)$$

where N = the number of fish, x = age of fish, t = year, and $S_x$ = the age-specific survival rate.

We simulated four levels of annual mortality (20, 25, 30, and 35%) on gulf sturgeon of 35- to 75-cm total length. The range in annual mortality was selected based on simulations that produced populations similar in size and trajectory to those estimated from collection data[16]. The size range (35 to 75 cm) we used corresponds to juvenile gulf sturgeon ages 1 to 3 in the Suwannee River[12]. Life-history attributes other than annual juvenile mortality were held constant or within fixed ranges during all simulations in this study (Table 1). Each simulation was allowed to run for 200 years. We eliminated the initial 50-year period from simulations to ensure that changes to input parameters were the only influence in simulation outcomes.

**TABLE 1**
**Population Attributes Held Constant or within Set Ranges in MOCPOP Simulation Models**

| Variable | Value | Reference |
|---|---|---|
| von Bertalanffy growth equation | $L_{t(cm)} = 222.273[1-e^{-0.08042\ (t\ +\ -2.18)}]$ | 16 |
| Fork length-wt relationship | $Weight_g = 1 \times 10^{-6}\ L_{mm}^{3.26}$ | 16 |
| Female age at first maturity | Age = 10 | 6 |
| Percent population that is female | 50% | 6 |
| % Females that spawn annually | 5% | 12,16 |
| Fork length-fecundity parameters | $F = 3.39 \times 10^{-4}\ L_{cm}^{4.05}$ | 15 |
| Beverton-Holt density-dependent relationship between reproductive potential and realized egg deposition | A = 0.2<br>Replacement ($P_r$) = 2,370,343 | 12,14,16 |
| Mortality | Egg to Age 1 random between (0.9996 - 1.0) | 16 |
| | Annual %<br>age 4 to 25 = 16% | 12,16,19 |

Modified from Pine et al.[16]

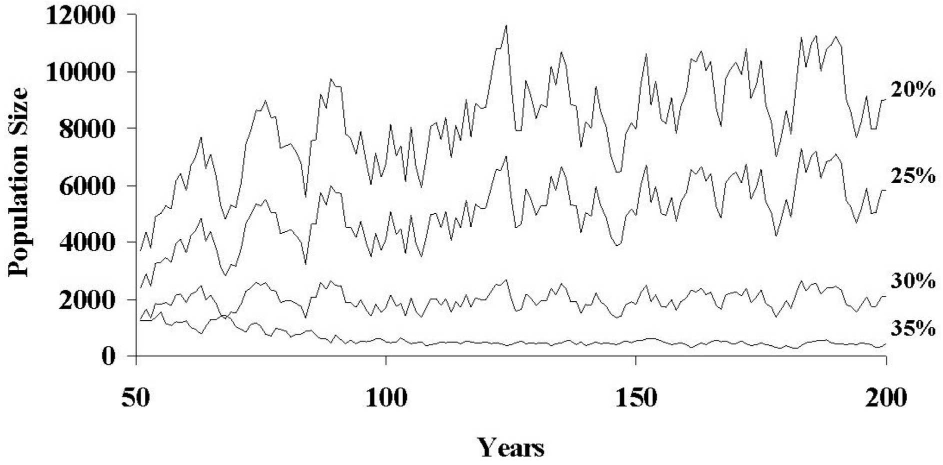

**FIGURE 1**. Simulated population responses to varied mortality rates on gulf sturgeon between 35- and 150-cm total length, using an age-structured model. Populations were simulated for 200 years, dropping the initial 50 years to ensure that changes to input parameters were the only influence in simulation outcomes. Variables other than mortality were held constant or within set ranges.

## RESULTS

As the annual mortality rate of juvenile gulf sturgeon increased, the trajectories of the simulated populations shifted from increasing to decreasing. At 20% annual juvenile mortality, the simulated population increased in size, to fluctuate between 8,000 and 10,000 individuals (Fig. 1). The simulation with a juvenile mortality rate of 25% increased to a population size between 5,000 and 6,000 individuals over 200 years (Fig. 1). Both of these populations appeared to be steadily increasing at the end of the simulated time period. Increasing juvenile mortality to 30% caused the simulated population to be around 2,000 individuals with a nearly stable trajectory. A 35% annual juvenile mortality rate resulted in gradual population decline throughout the simulated time period, to a population size near 200 individuals, and a continuing trend towards population collapse (Fig. 1).

## DISCUSSION

Our models indicated that even small increases in mortality of fish between 35 and 75 cm may have relatively large negative impacts on the population viability of gulf sturgeon. As the annual rate of juvenile mortality was increased from 20 to 35%, population sizes were reduced, and trajectories shifted from increasing to decreasing. When mortality was set at 20 or 25% annually, the simulated populations were observed to increase in size, and continued population growth would be expected. At a mortality rate of 30%, the simulated population was unable to increase in size, though it did appear to be stable. A juvenile mortality rate of 35% produced a population that was not viable.

The Suwannee River population has historically supported the largest and most stable gulf sturgeon population[8]. Carr et al.[10] estimated the size of this population to be between 1,504 and 3,066 individuals. Chapman et al.[17] provided estimates ranging from 2,097 to 5,312, and Sulak and Clugston[12] estimated the population to be 7,650 individuals. Other populations, however, are not as large or stable as the Suwannee River. For example, population size is much lower for gulf sturgeon populations in the Apalachicola[18] and Pearl Rivers, Florida[19]. In the Apalachicola River, Zehfuss et al.[18] estimated the population of gulf sturgeon to be between 62 and 218 individuals and indicated that the actual population is probably near 100. Similarly, the

Pearl River population was estimated to be 292 individuals[19]. Thus, increases in juvenile mortality in these populations may pose a greater threat to population viability than the Suwannee River population we simulated. Unlike the Suwannee River, the estuaries at the mouth of the Apalachicola and Pearl Rivers also support large commercial shrimp fisheries, where gulf sturgeon may be incidentally captured as bycatch[9]. Even when turtle excluder devices (TEDs) or bycatch reduction devices (BRDs) are used properly, commercial bycatch could potentially account for substantial mortality of juvenile gulf sturgeon. Our simulations indicated that population viability was highly sensitive to changes in juvenile mortality. Thus, bycatch and subsequent mortality of juvenile gulf sturgeon due to commercial fisheries may have potentially large impacts on gulf sturgeon populations. Unfortunately, estimates of bycatch and resulting gulf sturgeon mortality are not known, but should be evaluated.

Juvenile survival is important to population growth of long-lived marine species[1,20], and mortality due to bycatch in commercial fisheries may have large effects on the health of these populations[21]. Our results indicate this is also true for gulf sturgeon, though models could be enhanced if juvenile mortality were measured. Pine et al.[16] extrapolated juvenile mortality from published mortality estimates of adults in the Suwannee River and other populations of gulf sturgeon; whereas we varied juvenile mortality in simulations of the Suwannee River population. However, true estimates of juvenile gulf sturgeon mortality based on field studies have not been determined. We have illustrated the importance of juvenile survival to gulf sturgeon populations, which will hopefully prompt life-history studies in estuarine and marine habitats. Information gained from these studies may serve to further enhance demographic models and recovery efforts for gulf sturgeon and may be applied to other long-lived marine species.

## References

1. Crouse, D.T., Crowder, L.B., and Caswell, H. (1987) A stage-based population model for loggerhead sea turtles and implications for conservation. *Ecology* **68**, 1412–1423.
2. Cortes, E. (1998) Demographic analysis as an aid in shark stock assessment and management. *Fish. Res.* **39**, 199–208.
3. Musick, J.A. (1999) Ecology and conservation of long-lived marine animals. In Life in the Slow Lane: Ecology and Conservation of Long-Lived Marine Animals. Musick, J.A., Ed., American Fisheries Society Symposium 23. Bethesda, MD. pp. 1–10.
4. Sedberry, G.R., Andrade, C.A.P., Carlin, J.L., Chapman, R.W., Luckhurst, B.E., Manooch, C.S., Menezes, G., Thomsen, B., and Ulrich, G.F. (1999) Wreckfish *Polyprion americanus* in the North Atlantic: fisheries, biology, and management of a widely distributed and long-lived fish. In Life in the Slow Lane: Ecology and Conservation of Long-Lived Marine Animals. Musick, J.A., Ed., American Fisheries Society Symposium 23, Bethesda, MD. pp. 27–50.
5. Marmontel, M., Humphrey, S.R., and O'Shea, T.J. (1997) Population viability analysis of the Florida manatee (*Trichechus manatus latirostris*), 1976–1991. *Conserv. Biol*. **11**, 467–481.
6. Huff, J.A. (1975) Life History of the Gulf of Mexico Sturgeon *Acipenser oxyrhinchus de sotoi* in the Suwannee River, Florida. Marine Resources Publication 16. Florida Department of Natural Resources, St. Petersburg, FL.
7. Odenkirk, J.S. (1991) Movements of Gulf of Mexico sturgeon in the Apalachicola River, Florida. In Proceedings of the Annual Conference of SEAFWA. Vol. 43. pp. 230–238.
8. U.S. Fish and Wildlife Service and Gulf States Marine Fisheries Commission. (1995) Gulf Sturgeon Recovery Plan. Atlanta, GA. 170 p.
9. Wooley, C.M. and Crateau, E.J. (1982) Observations of Gulf of Mexico sturgeon (*Acipenser oxyrhinchus de sotoi*) in the Apalachicola River, Florida. *Fla. Sci.* **45**, 244–248.
10. Carr, S.H., Tatman, F., and Chapman, F.A. (1996) Observations on the natural history of the Gulf of Mexico sturgeon (*Acipenser oxyrhinchus de sotoi* Vladykov 1955) in the Suwannee River, southeastern United States. *Ecol. Fresh. Fish* **5**, 169–174.
11. Foster, A.M. and Clugston, J.P. (1997) Seasonal migration of gulf sturgeon in the Suwannee River, Florida. *Trans. Am. Fish. Soc.* **126**, 302–308.
12. Sulak, K.J. and Clugston, J.P. (1999) Recent advances in life history of Gulf of Mexico sturgeon *Acipenser oxyrinchus de sotoi* in the Suwannee River, Florida, USA: a synopsis. *J. Appl. Ichthyol*. **15**, 116–128.

13. Fox, D.A., Hightower, J.E., and Parauka, F.M. (2000) Gulf sturgeon spawning migration and habitat in the Choctawhatchee River system, Alabama-Florida. *Trans. Am. Fish. Soc*. **129**, 811–826.
14. Beamesderfer, R.C. (1991) MOCPOP 2.0: a flexible system for simulation of age-structured populations and stock-related functions. Oregon Department of Fish and Wildlife, Information Report 91-4, Portland, OR.
15. Beamesderfer, R.C., Rein, T.A., and Nigro, A.A. (1995) Differences in the dynamics and potential production of impounded and unimpounded white sturgeon populations in the lower Columbia River. *Trans. Am. Fish. Soc*. **124**, 857–872.
16. Pine, W.E., Allen, M.S., and Dreitz, V.J. (2001) Population viability of the Gulf of Mexico sturgeon in the Suwannee River, Florida. *Trans. Am. Fish. Soc*. **130**, 1170–1180.
17. Chapman, F.A., Hartless, C.S., and Carr, S.H. (1997) Population size estimates of sturgeon in the Suwannee River, Florida, U.S.A. *Gulf of Mexico Sci.* **2**, 88–91.
18. Zehfuss, K.P., Hightower, J.E., and Pollock, K.H. ( 1999) Abundance of gulf sturgeon in the Apalachicola River, Florida. *Trans. Am. Fish. Soc*. **128**, 130–143.
19. Morrow, J.V. Jr., Kirk, J.P., Killgore, K.J., Rogillio, H., and Knight, C. (1998) Status and recovery potential of Gulf sturgeon in the Pearl River system, Louisiana-Mississippi. *N. Am. J. Fish. Man*. **18**, 798–808.
20. Heppell, S.S., Crowder, L.B., and Menzel, T.R. (1999) Life table analysis of long-lived marine species with implications for conservation and management. In Life in the Slow Lane: Ecology and Conservation of Long-Lived Marine Animals. Musick, J.A., Ed. American Fisheries Society Symposium 23, Bethesda, MD. pp. 137–148.
21. Secor, D.H., and Waldman, J.R. (1999) Historical abundance of Delaware Bay Atlantic sturgeon and potential rate of recovery. In Life in the Slow Lane: Ecology and Conservation of Long-Lived Marine Animals. Musick, J.A., Ed. American Fisheries Society Symposium 23, Bethesda, MD. pp. 203–216.
